# Occipital artery to vertebral artery bypass for treatment of bilateral vertebral artery occlusion with QMRA as an adjunct to diagnostic assessment

**DOI:** 10.1007/s00701-024-06099-7

**Published:** 2024-05-07

**Authors:** Hynek Zitek, Ales Hejcl, Morteza Sadeh, Fady T. Charbel, Martin Sames

**Affiliations:** 1 Department of Neurosurgery, Purkinje University, Masaryk Hospital, Usti nad Labem, Czech Republic; 2 Department of Neurological Surgery, University of Illinois at Chicago, Chicago, IL USA

**Keywords:** Vertebrobasilar insufficiency, Vertebral artery, Occipital artery, Flow augmentation, Quantitative magnetic resonance angiography

## Abstract

**Purpose:**

Stroke, the second leading cause of death globally, often involves ischemia in the vertebrobasilar territory. This condition is underexplored, despite significant morbidity and mortality risks. The purpose of this study is to present a case of occipital artery to V3 segment vertebral artery bypass, emphasizing the role of quantitative magnetic resonance angiography (qMRA) in assessing flow and guiding surgical intervention.

**Methods:**

A 66-year-old man with bilateral vertebral artery occlusion presented acute symptoms. qMRA was employed to evaluate flow dynamics and determine the feasibility of a flow augmentation bypass surgery. The occipital artery to left vertebral artery bypass (OA-to-VA) was performed, utilizing an inverted hockey-stick incision and an antegrade inside-out technique. The patency of the bypass was confirmed using both Doppler probe and Indocyanine green.

**Results:**

Postoperative assessments, including computed tomography angiography (CTA) and qMRA, demonstrated the patency of the bypass with improved flow in the basilar artery and left vertebral artery. The patient's condition remained stable postoperatively, with residual peripheral palsy of the left facial nerve.

**Conclusion:**

In conclusion, the presented case illustrates the efficacy of the OA-to-VA bypass in addressing symptomatic bilateral vertebral artery occlusion. The study underscores the pivotal role of qMRA in pre- and postoperative assessments, providing noninvasive flow quantification for diagnostic considerations and long-term follow-up in patients with vertebrobasilar insufficiency.

**Supplementary Information:**

The online version contains supplementary material available at 10.1007/s00701-024-06099-7.

## Introduction

Stroke is the second most common cause of death worldwide. Approximately 87% of strokes are ischemic, mostly involving the carotid territory, and about 20–30% of ischemic strokes occur in the vertebrobasilar territory. This aetiology of vertebrobasilar stroke is often overlooked, despite the fact that it often affects brainstem function and is associated with significant morbidity or mortality in up to 20% of cases [10]. According to the New England Medical Center Posterior Registry, hemodynamically significant steno-occlusive disease of the great artery of the posterior circulation is responsible for 32% of strokes [7]. Moreover, those who suffer from posterior circulation stroke have 15% risk of recurrence despite maximal medical therapy [8]. Historically, for various reasons, ischemia in the posterior teritorry has not received as much attention as in the anterior circulation. One of the contributing factors is the more challenging hemodynamic evaluation of the posterior circulation vessels mainly due to anatomical constraints. The use of quantitative magnetic resonance angiography (qMRA) coupled with noninvasive optimal vessel analysis (NOVA) *VasSol, Inc, River Forest, IL. USA*. made a significant contribution to hemodynamic investigations in the posterior circulation and better understanding of the disease process affecting this particular area.

We present a case of occipital artery to V3 segment vertebral artery bypass in a patient with symptomatic bilateral vertebral artery occlusion and retrograde flow in the basilar artery. qMRA was used to determine the indication for a flow augmentation bypass surgery, and evaluate the hemodynamic effect of flow augmentation after bypass surgery.

## Technique and case illustration

**Fig. 1:**
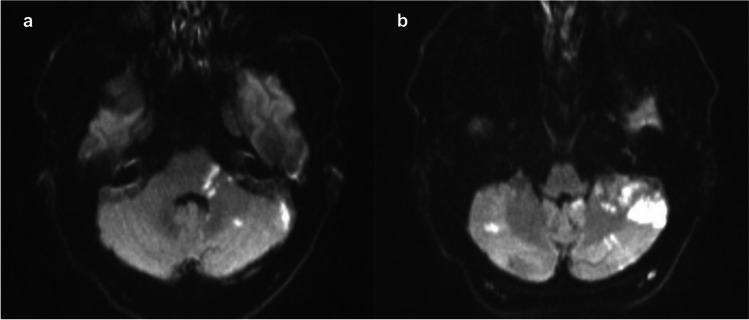
Showing several ischemic lesions in both superior cerebellar artery (**a**) and posterior inferior cerebellar artery (**b**) territories

A 66-year-old man with medical history of arterial hypertension and hyperlipidemia presented with acute onset of vertigo, diplopia, nystagmus in both horizontal and vertical directions, as well as peripheral palsy of the left facial nerve. Computed tomography angiography (CTA) at admission demonstrated occlusion of both vertebral arteries at V1 segments, both V3 segments were partially filled by collateral contribution. Diffusion-weighted MRI scans showed several diffusion restricted areas in both posterior inferior cerebellar artery territories and left superior cerebellar artery territory (Fig. 1).

**Fig. 2:**
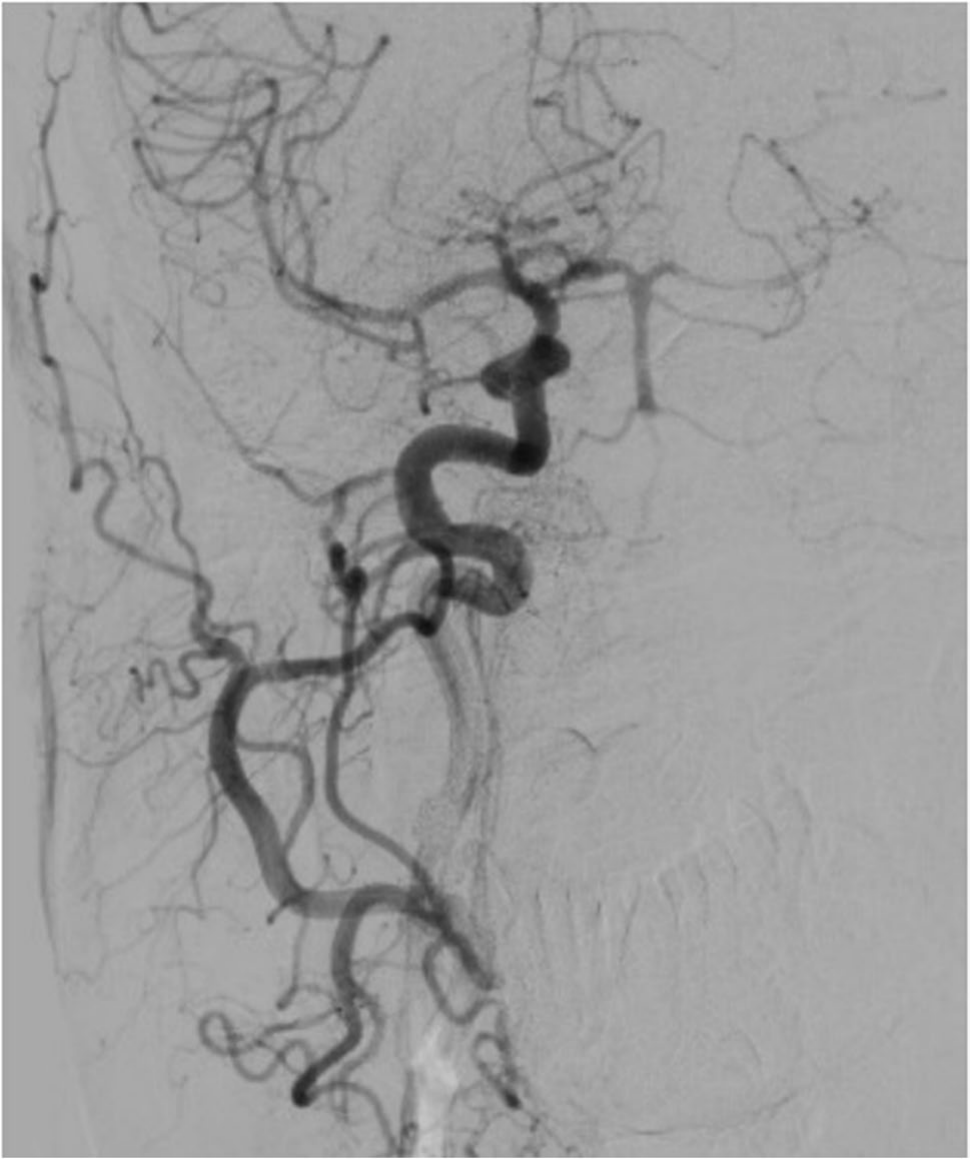
Digital subtraction angiography (DSA) picture showing filling of the distal posterior circulation up to V3 segments of vertebral arteries mainly through right posterior communicating artery

**Fig. 3:**
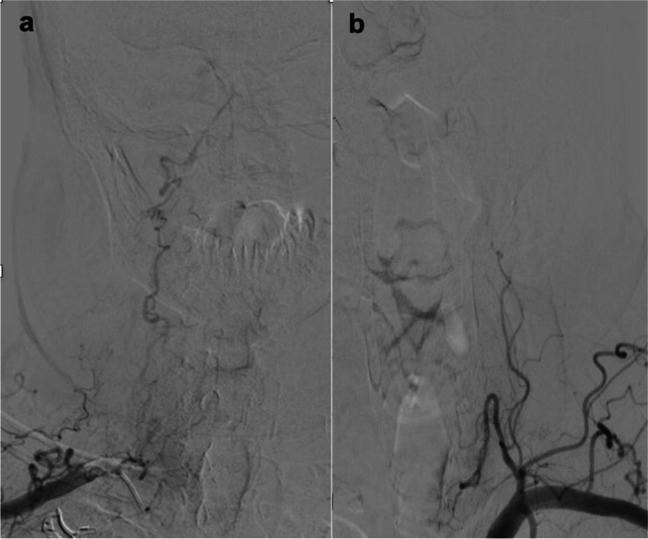
DSA of both subclavian arteries showing the right VA occlusion and a collateral from the right thyrocervical trunk filling the right VA territory up to the right PICA (**a**). There are no collaterals on the left side (**b**)

Digital subtration angiography (DSA) demonstrated filling of the distal posterior circulation up to V3 segments of vertebral arteries mainly through right posterior communicating artery (Fig. 2). DSA of subclavian arteries showed a collateral from the right thyrocervical trunk filling right VA up to right PICA and no collaterals on the left side (Fig. 3).

**Fig. 4:**
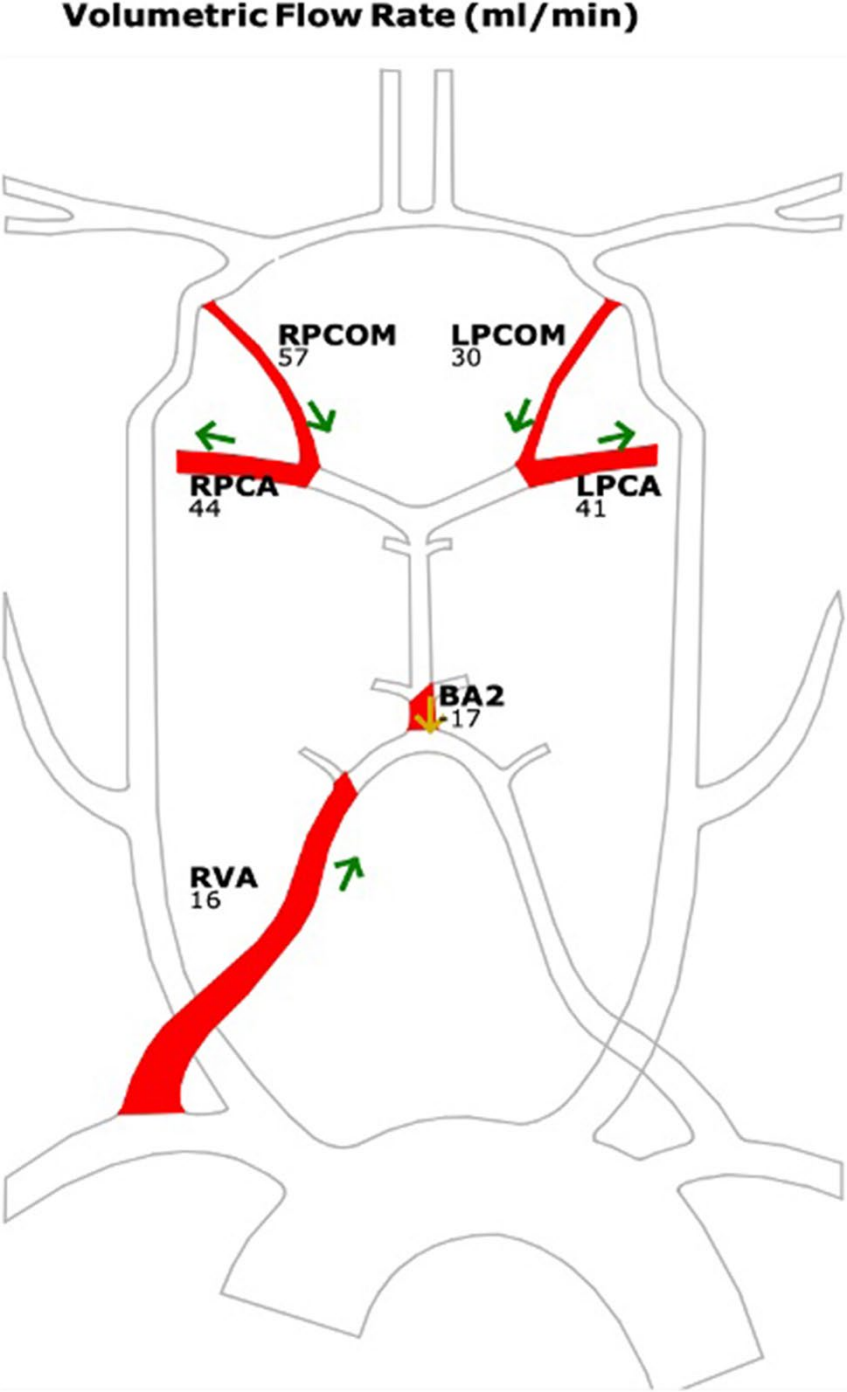
QMRA showing low and reversed flow in basilar artery (17 ml/min), retrograde flow in both PComs (30 and 57 ml/min). This means distal low flow and high-risk patient

Perfusion CT demonstrated hypoperfusion of left PICA territory with lower cerebral blod flow (CBF) and longer mean transit time (MTT). QMRA showed low and reversed flow in basilar artery (17 ml/min), retrograde flow in both PComs (30 and 57 ml/min) (Fig. 4).

**Fig. 5:**
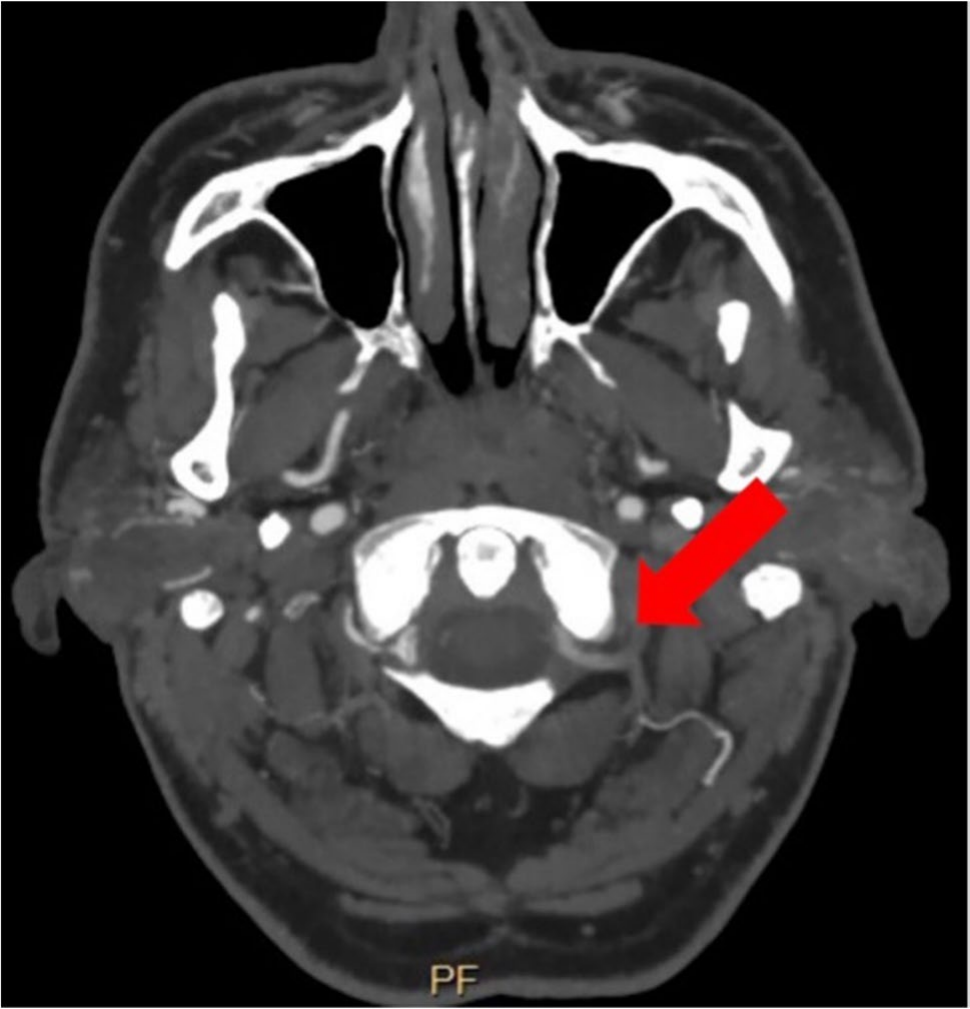
CT angiography proving good patency of V3 segment on the left side

QMRA confirmed diminished perfusion of the posterior fossa with elevated risk of recurrent stroke. EC-IC bypass for flow augmentation was recommended. We decided on the horizontal part of V3 segment of the left vertebral artery as the recepient artery since the retrograde flow was preserved in this segment (Fig. 5).

**Fig. 6:**
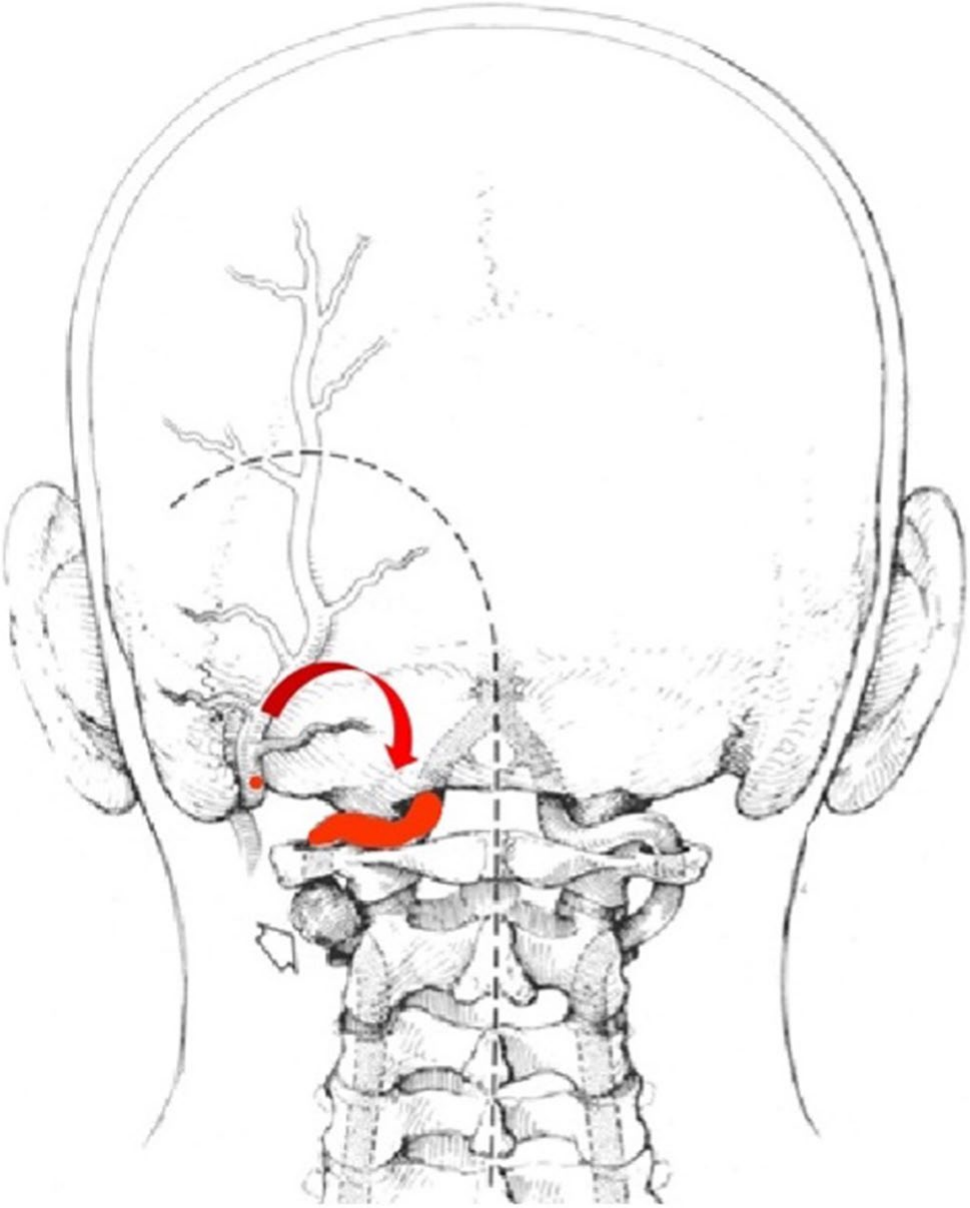
Schematic drawing showing anatomical principles of OA to eVA bypass

We therefore approached the bypass via an occipital artery to left vertebral artery technique (OA-to-VA bypass). This bypass did not require craniotomy with durotomy since V3 segment is accessible extracranially. Patient was positioned prone in Mayfield head holder. An inverted hockey-stick incision from the spinous process of C2 to the mastoid tip was made and the occipital artery was identified in the occipital groove just medial to the digastric groove. The antegrade inside-out technique according to Benet et al. was used [4] (Fig. 6).

**Fig. 7:**
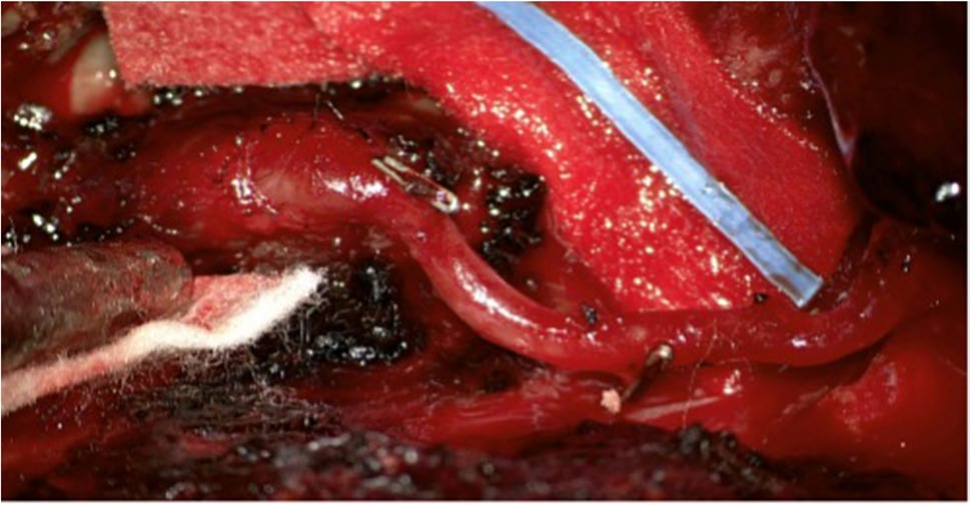
Showing completed bypass of OA to eVA

The left part of the posterior arch of C1 below horizontal segment of V3 was partially drilled and the recipient artery was identified and dissected from the surrounding tissue. We perfomed end to side anastomosis of the OA to V3 segment using interrupted sutures and fish-mouth method for donor vessel (Fig. 7).

The patency of the bypass was confirmed using both Doppler probe and Indocyanine green. The procedure and postoperative period were uneventful. (The whole procedure is summarized in Video 1 in supplementary material.)

**Fig. 8:**
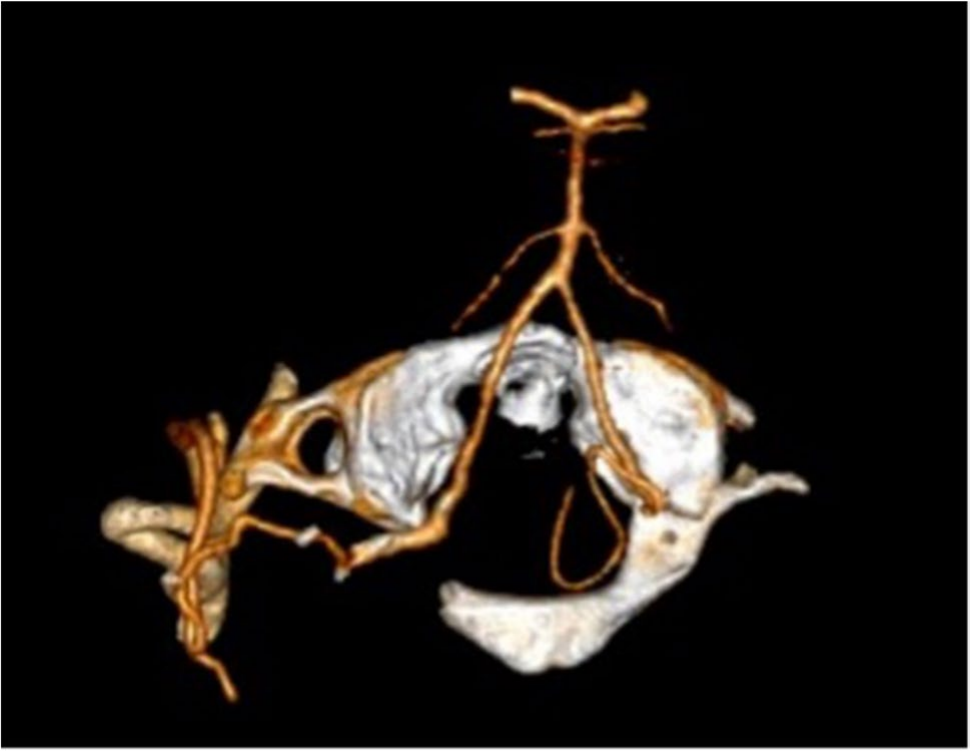
Postoperative CT angiography showing good patency of the bypass and partial removal of the posterior arch of C1 in order to expose the recipient artery

The postoperative CTA demonstrated patency of the bypass (Fig. 8).

**Fig. 9:**
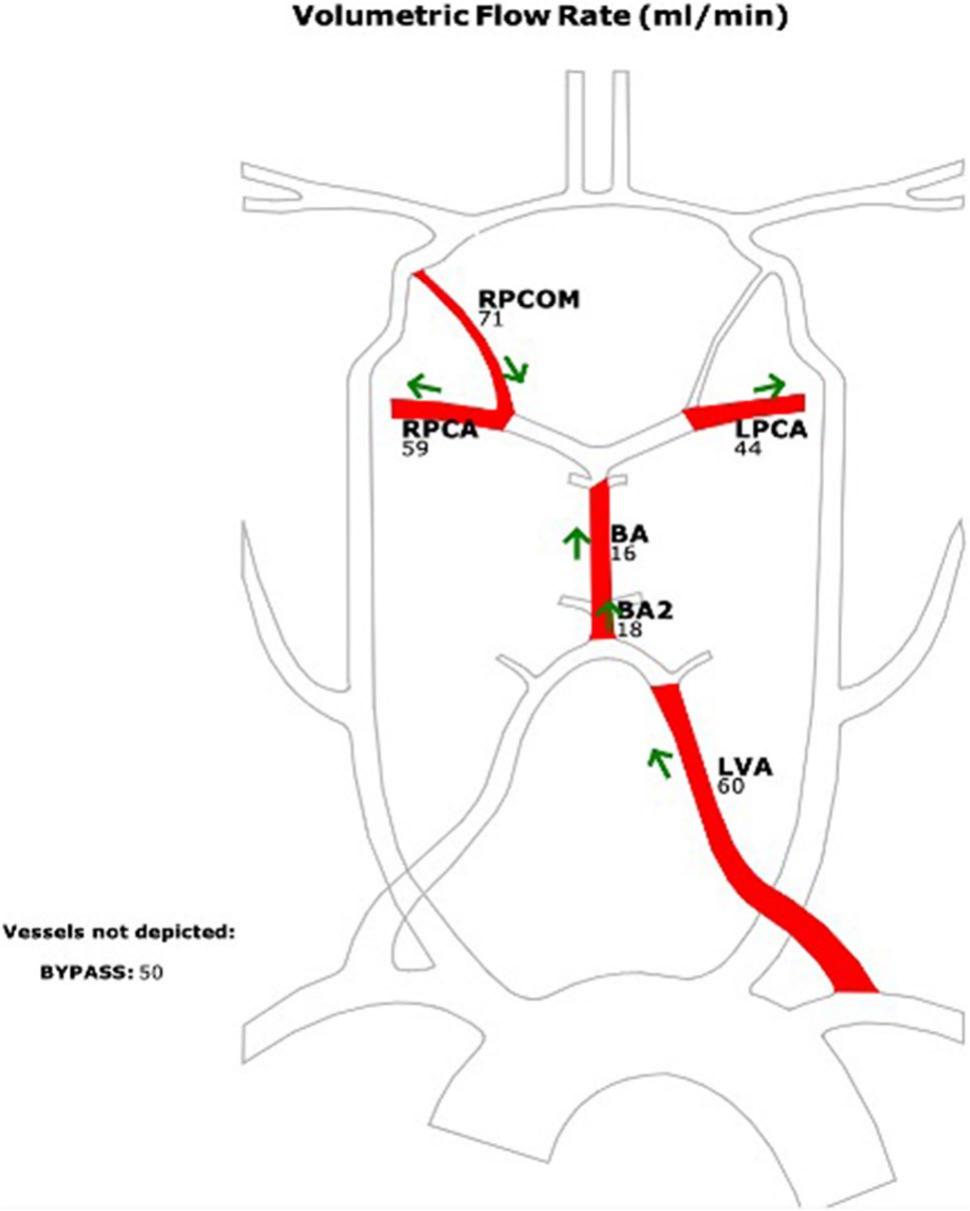
QMRA showing change in direction of flow in the basilar artery after bypass (orthograde 16 to 18 ml/min). Bypass flow was 50 ml/min

Postoperative QMRA showed excellent revascularization of the territory with reversal of blood flow in the basilar artery and left vertebral artery, the flow was also increased in both PCAs (Fig. 9).

The patient conditon has been stable since the procedure with residual peripheral palsy of the left facial nerve. The ischemic event has not recurred.

## Discussion

We present a rare case of successful surgical revascularization of the posterior circulation in a patient with bilateral vertebral artery occlusion. Although ischemia in the vertebrobasilar territory accounts for a smaller proportion compared to anterior circulation stroke, it should not be overlooked and should be throughly investigated when suspected. First, because it supplies highly vulnerable brainstem and thalamic areas and also because symptomatic vertebral artery stenosis has a higher 90-day recurrence risk than symptomatic carotid artery stenosis, reaching up to 22% despite maximal medical therapy [1, 15]. In the case of bilateral vertebral artery occlusion, the prognosis of patients is not precisely known, but it is certainly abysmal despite medical therapy [6]. The role of endovascular treatment in secondary prophylaxis in these patients is controversial since 2 randomized controlled trials (RCT) of VIST and VAST failed to show benefit in patients with extra and intracranial vertebral stenosis in comparison to medical therapy [9, 16]. The value of surgical intervention also remains unclear because of insufficient evidence for posterior revascularization techniques [14]. However, for patients with recurrent symptoms with stenosis or occlusion causing vertebrobasilar insufficiency, it may be a last resort for rescue and should be entertained as an option. The use of QMRA NOVA to quantify flow in the main arteries and differentiate patients with low distal flow has signifcantly facilitated the identifcation of patients who may benefit from posterior circulation revascularization. These patients have either lower flow in the basilar artery than 120 ml/min or lower flow in both posterior cerebral arteries than 40 ml/min [2]. The follow-up prospective, blinded VERiTAS Study demonstrated a significant association between patients with low distal flow and the risk of recurrent stroke in patients with symptomatic macroangiopathy in the vertebrobasilar territory, with an event-free survival rate at 24 months of 70% in low-flow group vs 87% in normal-flow group [3]. Furthermore, in 2020, an optimization of the protocol was published by use of vessel anatomy (fetal PCA) and age-specific normalized flows [17]. In our case we used QMRA to verify diminished flow in the major vessels of the posterior circulation. The reversed flow in both PCAs was not able to adequately replace the flow into the vertebrobasilar area, and flow values in both PCAs and BA confirmed low distal flow. In conjunction with the findings on CT angiography, DSA and CT perfusion, EC-IC bypass was indicated.

Several surgical revascularization techniques for posterior circulation have been described. The first EC-IC bypass was performer by Khodah et al. (1976) who used OA to posterior inferior cerebellar artery (PICA) anastomosis [13]. This bypass technique remains perhaps the most popular for this indication to this day. Other routes include the superficial temporal artery to superior cerebellar artery (STA-SCA), STA to posterior cerebral artery (PCA) [11]. Some authors used interposition graft from radial artery or saphenous vein to anastomose carotid artery with the PCA [5, 19]. These aforementioned techniques have been proved functional but many of them demand complex surgical approaches with longer surgical times and higher probability of surgical complications. We decided to take advantage of the the preserved patency of the V3 segment of the left vertebral artery according to DSA and performed occipital artery (OA) to extradural vertebral artery (eVA) bypass. This technique was first described by Hadley et al. (1985) as a flow replacement for clipping of traumatic pseudoaneurysms. In the same article, the authors mentioned the use of the same technique in two patients with thrombo-occlusive vertebrobasilar disease [11]. We chose hockey-stick incision with inside-out harvesting technique of the vertebral artery according to Benet et al. [4]. We opted for this technique for its relative simplicity and straightforward approach of access to occipital artery with avoidance of tedious layer-by-layer dissection of suboccipital muscles. There are several advantages of performing an extradural bypass if it is feasible. These include avoidance of the need for craniotomy, absence of durotomy and thus minimizing the risk of CSF leakage, and a relatively shallow operative field. However, if necessary, direct extension of this approach allows intradural inspection of the vertebral artery. In 2018, Katsuki et al. published their case of OA to eVA bypass for bilateral symptomatic vertebral artery stenosis with good graphic and clinical outcome. They also found only 19 other published cases of the same bypass with results either not described or with good patency and no complications until then [12]. Then in 2019, Wang et. al. published a series of 17 patients with OA to eVA bypass with posterior circulation ischemia with 100% early postoperative patency of the bypass according to DSA. Good long-term clinical outcome (modified Rankin score 0–2) was described in 82% of patients. There was one recurrent stroke with bypass occlusion [20].

The benefit of QMRA is also considerable postoperatively, as it offers valuable information regarding the change in flow direction and its quantity. It is thus an important complement to anatomical examinations such as CT angiography and DSA. Change in flow direction and change in flow magnitude can also help in predicting the demand in a given region and hence predict the safety and patency of bypass surgery [2, 18]. In our case, there was a change in direction in the BA and improvement in flow in both PCAs, and thus, good long-term patency of the bypass performed can be expected.

## Conclusion

Our case of OA to eVA bypass in a patient with symptomatic occlusion of both vertebral arteries illustrates this technique as a therapeutic alternative in patients with this severe condition. We also aim to highlight the important role of QMRA in pre- and postoperative assessment in these patients. This examination offers noninvasive quantification of flow in the major arteries of the vertebrobasilar territory and is a valuable adjunct in the diagnostic and indicative considerations, as well as in long-term postoperative follow-up in patients with vertebrobasilar insufficiency.

## Supplementary Information

Below is the link to the electronic supplementary material.

Supplementary file1 (MP4 63571 KB)

## Data Availability

The data that support the findings of this study are available from the corresponding author (H.Z.) upon reasonable request.
